# Atlanto-Axial Instability in People with Down’s Syndrome and its Impact on the Ability to Perform Sports Activities – A Review

**DOI:** 10.1515/hukin-2015-0087

**Published:** 2015-01-12

**Authors:** Andrzej Myśliwiec, Adam Posłuszny, Edward Saulicz, Iwona Doroniewicz, Paweł Linek, Tomasz Wolny, Andrzej Knapik, Jerzy Rottermund, Piotr Żmijewski, Paweł Cieszczyk

**Affiliations:** 1 Department of Kinesitherapy and Special Methods in Physiotherapy, The Jerzy Kukuczka Academy of Physical Education, Katowice, Poland; 2 The Joseph Tischner Special Schools Complex no.10 in Jastrzębie-Zdrój, Poland; 3 The Academy of Business in Dąbrowa Górnicza, Poland; 4 School of Health Sciences in Katowice, Medical University of Silesia, Department of Rehabilitation; 5 Department of Health Care, Medical University of Silesia in Katowice, Poland; 6 Department of Physiotherapy, Higher School of Administration in Bielsko Biała, Poland; 7 Department of Physiology, Institute of Sport, Warsaw Poland; 8 Faculty of Physical Education and Health Promotion, University of Szczecin, Poland

**Keywords:** atlanto-axial instability, Down’s syndrome, spinal cord compression, medical imaging

## Abstract

Atlanto-axial instability (AAI) is a developmental anomaly often occurring in persons with Down’s syndrome (DS). According to various reports, AAI affects from 6.8 to 27% of the population with DS. The aim of this review was to illustrate the issue of AAI with regard to the progressively changing state of scientific knowledge. The extended distance between the rear surface of the frontal arcus of the C1 cervical vertebra and the anterior surface of C2 cervical vertebra dens (anterior atlanto-odontoid distance, AAOD) indicates the occurrence of AAI and is detectable through X-ray examination. Hypoplasia of the C2 dens, also detectable through X-ray examination, is another suggested risk factor for AAI. According to current data, the methodology of taking measurements is inconsistent, which leads to errors in interpretation. As research focusing on AAI was progressing, new data emerged from other studies on persons with DS, suggesting that neurological symptoms in persons with DS that indicated the occurrence of spinal cord compression were an important factor in medical imaging detection of AAI. One of the main arguments supporting this thesis is that in isolated cases spinal cord (SC) damage was noted during screening examinations conducted on a large population of subjects. Moreover, cases in which the neurological symptoms indicate spinal cord compression existed long before the occurrence of the actual damage also remain of significant importance. Therefore, it is necessary to promote neurological studies on persons with DS to enable early diagnosis of spinal cord compression and, at the same time, reduce the use of medical imaging in cases of neurological symptoms.

## Introduction

Atlanto-axial instability (AAI) is a developmental anomaly often occurring in people with Down’s syndrome (DS). According to various reports, AAI affects from 6.8 to 27% of the population with DS.

The extended distance between the rear surface of the frontal arcus of the C1 cervical vertebra and the anterior surface of C2 cervical vertebra dens (anterior atlanto-odontoid distance, AAOD) indicates the occurrence of AAI. During X-ray examination, the distance is measured in a neutral position with forward flexion of the cervical spine and, as suggested by some reports, in a position of hyperextension (Ferguson et al., 1997; Harley and Collins, 1994; Kattan and McDonald, 1996; Roy et al., 1993).

AAI was first mentioned in ancient times (Pueschel, 1992a). The first screening examinations were conducted in the 1960s by Spitzer et al. (1961), as well as Tishler and Martel (1965).

A rise of interest on AAI was noted in 1984, when the American Academy of Pediatrics Sports Committee supported the initiative of the Special Olympics Committee regarding the necessity of performing radiological examinations on children with DS. The aim was to prevent potential spinal cord injuries during sports and recreational activities (American Academy of Pediatrics, 1984; Msall et al., 1990; Selby et al., 1991). The rules of the Special Olympics require every athlete to undergo radiological examination. If the physician diagnoses the athlete with AAI, then the athlete is excluded from some of the events – butterfly style swimming, starting block dives, jumping into the water, pentathlon, a high jump, horseback riding, gymnastics, football, barbell squat in powerlifting, downhill skiing as well as warm up exercises taxing the head and the neck (Official Special Olympics Rules). The limits set by the official rules remain valid, although in 1995, after reviewing current studies on AAI, the American Academy of Pediatrics withdrew the rule obliging every athlete with DS to undergo radiological examinations. At the same time, more and more opinions emerged stating that the factual threat to life and health of persons with AAI, as well as the percentage of people with AAI suffering from spinal cord damage, was insignificant. Thereafter, it was suggested to accentuate the need for performing neurological examinations to evaluate the level of spinal cord compression and to conduct information campaigns regarding the recognition of early symptoms of spinal cord compression among people with DS. The aim of the information campaigns was for the families of the people with DS to be able to recognize the symptoms and to refer the patients to specialists for examination.

The purpose of this review was to analyse the current data on AAI in people with DS and to summarize the current state of knowledge on this topic. Moreover, the secondary objective of this review was to prepare the ground for discussion that would involve those associated with Special Olympics Poland, as well as to view the potential options for change in the Special Olympics regulations.

## Material and Methods

To select articles on the subject of AAI in people with DS, we searched PubMed, ScienceDirect and EBSCO electronic databases. The key words used for searches were *atlanto-axial instability, atlantoaxial instability, atlantoaxial dislocation, atlantoaxial anomaly, atlantodental instability, atlantodental dislocation, atlantodental anomaly, Down syndrome, Down’s syndrome,* and *21 chromosome trisomy.*

Depending on the database, various amounts of matching records were found: PubMed, approximately 100; ScienceDirect, close to 400; EBSCO, near 160. However, most of the records concerned the treatment of the atlanto-axial joint from a neurological, orthopaedic, or surgical perspective and did not relate to the subject of rehabilitation for physical and sports activities in people with DS. After filtering the content to eliminate repeated records, 40 publications remained, which were then thoroughly analysed with regard to methods of taking measurements, causes and effects of AAI in physically active people with DS in the context of the constantly altering views on the subject.

### History

Anomalies of the cervical spine were first noted in ancient Egypt about 4500 years ago. One discovered papyrus describes a case of a person with dislocated cervical vertebra. Obviously, the term “atlanto-axial instability” was not used at the time (Power, 1933).

In 1961, Robert Spitzer and his associates performed X-ray examinations of the skeletal system of 29 people with DS, aged 8 to 49. The results of the examinations showed that 9 people suffered from vertebra translocations within the atlanto-occipitalis joints and reduced height of the arcus of the C1 vertebra (Spitzer et al., 1961).

The first studies to take precise measurements of the AAOD were conducted in 1961 by Tishler and Martel. In a group of 18 people with DS, aged 2 to 56, 4 cases of the AAOD over 5 mm were noted (in a case of a 10-year-old girl, the distance amounted to 8 mm in a neutral position and 10 mm in flexion) and another 9 cases in which the distance amounted to 4 mm. However, at this stage the researchers were not able to provide an explanation regarding the cause of the AAOD anomaly occurring in so many patients with DS. Most studies on this topic were published in the 1980s and 1990s.

### Measurements

Recommendations for taking proper measurements of the AAOD and setting the limit values for AAI have been inconclusive since the very beginning. One group of researchers from the 1980s suggested that the upper limit of proper values of the AAOD should amount to 3.5 mm (White et al., 1975; Green et al., 1981) considering that a value of 3.5 mm or more indicated the occurrence of anomaly. Other researchers set the limit at 4 mm (Burke et al., 1985; Elliott et al., 1988, Jagjivan et al., 1988; Cremers et al., 1993). At the same time, the American Academy of Pediatrics (1984) suggested to set the upper limit of the AAOD at 4.5 mm. The very same criterion was suggested by Selby et al. (1991). Another group of researchers defined instability by the AAOD of 5 mm or more (Alvarez and Rubin, 1986; Pueschel et al., 1984, 1987; Smith, 2001). Seemingly minor differences between measurements (± 0.5 mm) are significant for people with DS who participate in sports and recreational activities. At the same time, as suggested by Selby et al. (1991), the credibility of X-ray examination measurements remains questionable. Selby and colleagues (1991) repeated the measurements of their patients after 10 minutes and again after three weeks from the first measurement. The results were diversified. Therefore, qualifying the participants of this study group as patients suffering from AAI or patients without the anomaly might have been accidental. They also observed that the emotional state of the patient actually affected the result of the examination with people in a calm and quiet mood showing greater symptoms of musculo-ligamentous hypotension, which may have affected the results of the AAOD measurement (i.e., greater AAOD). The tonicity was near normal in people who were excited or in a bad mood (Selby et al., 1991). The literature also mentions other examples of incorrectness regarding X-ray examination measurements such as improper calibration of the device, an improper position of the patient, and limited contact with the patient due to his or her cognitive disorders (Cremers et al., 1993; Elliott et al., 1988; Swinkels and Oostendorp, 1996).

The previously mentioned measurement limits also cannot be deemed absolute values. Therefore, regarding the dynamic character of stability and instability, the literature suggests that an additional qualification of the anomaly should be created, differentiating actual instability from subluxation. However, the views on this topic are contradictory. Green et al. (1981) suggested qualifying AAOD of 1–3 mm as subluxation, while AAOD of 3.5 mm or more should indicate instability. Morton et al. (1995) indicated, however, that the AAOD value of 1 mm in extension and 4 mm in flexion should be qualified as instability, while the AAOD of 5 mm, unaffected by extending or flexing, should be deemed subluxation.

Another matter referred to in the literature with regard to AAI measurements is the age of the patient. It has been noted that AAI has a tendency to decline with age. Therefore, the authors claim that the AAI standard should be revised. The authors suggest that in people aged less than 15, the AAOD of 4 mm or more should be considered symptomatic of AAI, while in persons aged 15 or above, the standard should be set at 3 mm (Burke et al., 1985; Jagjivan et al., 1988; Ali et al., 2006).

Although AAOD is an indicator of AAI used by researchers in numerous studies of the anomaly, there is also another distance that needs to be mentioned, as it is very important for describing the phenomenon of atlanto-axial joints instability. Ali et al. (2006) suggest the importance of measuring the distance between the rear surface of the C2 axis dens and the anterior surface of the rear arcus of the C1 vertebra (posterior atlanto-odontoid distance - PAOD). The authors noted that it is even a more significant indicator of AAI, supporting this thesis with an observation that in cases of dislocation between the C1 and C2 vertebras, the amount of space left for the spinal cord was of greater importance than the range of the dislocation. In physiological conditions, inflexion of the cervical spine requires that the PAOD should amount to more than 14 mm (Ali et al., 2006; El-Khouri et al., 2014).

### Causes

The causes of AAI are complex. It needs to be mentioned that despite its common occurrence in the population with DS, AAI also concerns other people. AAI symptoms are also related to post-tonsillectomy as well as post-cervical spine injury conditions, cases in which general anesthesia with intubation was used, juvenile rheumatoid arthritis, and degenerative states of the cervical vertebras (Ali et al., 2006; Dawidson, 1988; Swinkels and Oostendorp, 1996). It seems, however, that a frequent occurrence of AAI in people with DS comprises several factors working simultaneously and related to a set of risk factors. Birth defects within the musculoskeletal system, especially cervical spine defects, are obviously the main factors mentioned in the literature (Pueschel et al., 1990). The loosening of the transverse ligament of the C1 vertebra, which sets the limit of motion for the C2 axis dens in the sagittal plane, is another frequently mentioned cause. As suggested by the literature, the transverse ligament consists mainly of collagen fibers which in physiological conditions enable the ligament to endure heavy loads while remaining inflexible. Loosening of the transverse ligament may be related to chronic inflammation within the upper respiratory tract to which people with DS are susceptible due to breathing mainly through the mouth (Ali et al., 2006; Elliott et al., 1988b). Reduced muscle tension, especially in people with the so-called thyroid-type DS, may also contribute to overstraining the transverse ligament, as a result of reduced muscular stabilization of the cervical spine. C2 axis dens hypoplasia, occurring in people with DS nearly as often as AAI, may be another cause (Ali et al., 2006; Elliott, 1988a; Elliott et al., 1988b; Selby et al., 1990). Shortening the dens may facilitate the motion of the C1 transverse ligament over the peak of the dens and therefore increase the range of motion in the atlanto-axial joints in the forward direction.

## Results

Except for the unquestionable effect of instability due to overstraining of the facies articularis (this applies to any of the joints) and the occurrence of degeneration states or habitual subluxations as a result, the risk of spinal cord compression in the spinal canal remains of the greatest dangers related to AAI.

Unfortunately, as in the case of radiological examinations, the literature does not provide a unanimous opinion regarding the effect of AAI on the level of spinal cord damage or the credibility of the measurement methods.

The problem of AAI in people with SD gained recognition after the meeting of prominent American physicians, specialists in the field of sports medicine, and solicitors with the Special Olympics authorities in 1983. At that time about 500 000 people with SD in the United States practiced sports such as gymnastics, swimming, and other disciplines, which according to the participants of the meeting may have resulted in permanent disability in those subjects who suffered from both DS and AAI. The danger of spinal cord compression was accentuated at the meeting along with invoking some of the symptoms, such as gait disturbance, sphincter and bladder dysfunctions, pain and limited range of motion within the cervical spine, muscular fatigue, limb spasm symptoms, excessive tendon reflexes or muscle clonuses. At the same time, the low level of expertise on the topic among physicians was emphasized, given that the occurrence of AAI in people with DS was at the level of 12–15% of the population (Cooke, 1984). Due to the seriousness of the AAI issue, the authorities approved of the physicians recommendations regarding AAI and spread the topic of instability in people with DS among the communities of people with disability who were engaged in sports activities. At the same time, in 1984, the American Academy of Pediatrics Sports Committee supported the initiative of the Special Olympics regarding the necessity of performing radiological examinations in all children with DS (American Academy of Pediatrics, 1984; Cooke, 1984; Msall et al., 1990; Selby et al., 1991). The studies on AAI in relation to spinal cord compression have been conducted ever since in a number of different ways.

Some of the researchers note that each and every person with DS needs to undergo X-ray examination. These authors recognize the importance of analysing neurological symptoms to properly diagnose the potential compression of the spinal cord, also accentuating that some of the people with DS, due to cognitive disorders, as well as intellectual disability, may not be able to recognize the symptoms and therefore may not report them to their relatives (Cooke, 1984; Elliott et al., 1988b). Yet, another argument supporting the necessity to perform X-ray examinations is the possibility to prevent the development of AAI through proper surgical intervention before the symptoms occur; in addition, AAI is not a changeable type of anomaly, although the range of instability and its occurrence in general may change as the patient ages (Morton et al., 1995). To this day, undergoing X-ray examination remains obligatory for all Special Olympics athletes. The result of this examination determines the sport in which an athlete can participate (Special Olympics Official Rules for 2015).

Another group of researchers suggested that people with DS needed to undergo both radiological and X-ray examination simultaneously. The main argument for adopting such a protocol was the observation of a weak correlation between the occurrence of AAI and spinal cord compression symptoms. Due to low awareness of patients regarding the symptoms as well as limited ability to report the symptoms, it was suggested that both X-ray and neurological examinations should be performed in all children up to 6 years of age who qualified for medical or rehabilitation treatments. For children of 2 to 6 years of age, it was recommended that X-ray shots be taken before the medical or rehabilitation treatments in the following positions: neutral, flexion, and extension and before introducing a neurological examination protocol intended to become a part of a routine examination protocol for people with DS (Msall et al., 1990).

Interesting results were presented by Roya et al. (1990), who analysed the correlation between the occurrence of AAI and neurological symptoms, and indicated no correlation whatsoever. The authors noted that in case of many of the people with DS, the symptoms of spinal cord compression may be difficult to identify due to lack of physical activity considering that symptoms normally occur during physical activity. Nevertheless, undertaking sports activities or even recreational activities may pose a danger of a neurological symptoms flare-up.

Similar observations can be found in publications of Pueschel et al. (1981; 1990; 1992a; 1992b), who studied the topic of AAI in people with DS over several years. However, an in-depth analysis of Pueschel et al.’s work would transgress the topic of this article.

Over the course of time, as more studies on the occurrence of AAI in people with DS have been conducted, as well as the correlation between AAI and spinal cord compression, a new hypothesis represented by a large group of researchers has emerged, establishing a kind of compromise between previous views on the subject. The scientists emphasized a few issues related to AAI. Firstly, there was a lack of correlation between AAI and neurological symptoms, which supported the results of previous studies on the topic. Secondly, an unusually low number of cases of permanent and sudden damage to the spinal cord resulted from activities performed by the person with DS. Moreover, the potential neurological symptoms had existed long before the occurrence of sudden and permanent damage to the spinal cord, which created an early opportunity to implement proper treatment (Cremers et al., 1993a; El-Khouri et al., 2014; Wadhwa and Mummaneni, 2014). Selby et al. (1991) accentuated that the over-interpretation of radiological examination results posed numerous sports activity restrictions to people with DS, even though no symptoms of spinal cord compression could be found in those subjects.

On the other hand, based on the results of their experiment, Cremers et al. (1993b) suggested that sports performance by people with DS (both subjects with diagnosed AAI and those without AAI symptoms) did not affect the mobility of atlanto-axial joints in any way.

In 1995, the American Academy of Pediatrics withdrew previous recommendations, approving most of the arguments in favour of the necessity of performing neurological examinations, in particular.

More recent studies on the subject suggest no correlation between AAI and neurological symptoms (Ali et al., 2006; Dedlow et al., 2013; El-Khouri, 2014). Dedlow et al. (2013) even provided an example of a person with DS, who was unconfirmed for AAI during preschool years but later started showing neurological symptoms of spinal cord compression. The authors emphasized the importance of neurological examination, especially in people willing to engage in sports activities. Wadhwa and Mummaneni (2014), aside from the arguments listed above, also noted the necessity of educating the physicians and basic medical care staff on the topic of AAI and its proper diagnosing.

The results of the aforementioned studies, especially the current ones, criticize the results of previous research on AAI based on radiological examinations. It is also suggested that the diagnostics of the anomaly within the cervical spine need to be extended to neurological examination as well. Moreover, it is even implied that the neurological examination should serve as the basic examination as it is easy to perform, inexpensive, and, above all, eliminates the need to expose the patient to radiation.

The patient should be referred to the examination only after neurological symptoms occur to evaluate the range of the dislocation of upper cervical spine vertebrae and decide upon further treatment methods.

## Discussion

To summarize the analysis of the above-listed literature on the topic of AAI in people with DS, a few important conclusions need to be accentuated for the reader to comprehend the essence of the problem and to recollect the basic issues related to the occurrence of AAI. The observations made in this article are relevant both to people with DS as well as their communities, including family, health professionals (physicians, physiotherapists), and coaches.

The first remark involves the X-ray examination. The literature gives numerous examples of misconceptions regarding the conducting of X-ray examination. Both the process of taking X-ray photos as well as taking measurements have been criticized, which contributes to the low credibility of this method. Many authors also point to the harmfulness of radiation to the patient.

Numerous authors suggest a neurological examination as an alternative to X-ray examination as a basic method for detecting the dangers resulting from stability disorders within the C1 and C2 vertebra region. Multiple studies indicate the lack of correlation between the neurological and the X-ray examination as well as the dynamic character of the vertebra dislocations, which makes regular screening examinations obligatory, especially in people engaged in sports. A neurological examination, even when performed often, remains non-invasive and inexpensive, does not require costly medical devices, and is easy to perform for an experienced neurologist. Furthermore, neurological examinations are less stressful for the patient, which is a crucial factor regarding the emotional states of people with DS.

It is utterly important to organize information campaigns among people dealing with DS, including both health professionals and families of people with DS. Many scientists repeatedly accentuate the lack of awareness even among the so-called specialists regarding AAI and the neurological symptoms indicating spinal cord compression. Early detection of spinal cord compression symptoms may become crucial to adopting an optimal treatment protocol and preventing serious and permanent damage. Prevention plays an important role in the process, especially taking into account the cognitive disorders, intellectual disability, and communication limits the patients struggle with. However, caregivers and health care providers are often unaware of symptoms and problems which may consequently occur.

Finally, the limits set by the Special Olympics Official Rules with regard to people with DS and AAI participating in various sport disciplines are worth addressing. In accordance with the rules, the AAI evaluation consists of an X-ray examination in lateral projection, in a position of forward head flexion, and in a neutral position. Regarding the exquisitely rare cases of serious spinal cord damage resulting from sports activity in people with DS, the occurrence of spinal cord compression symptoms long before the injury, and the (repeatedly proven) lack of correlation between the symptoms of spinal cord compression and X-ray examination, it seems worthwhile for the Special Olympics authorities to revise the current rules involving people with DS. The potential exclusion of X-ray examinations and application of neurological examinations instead could eliminate the exposure to harmful radiation and reduce the costs of the evaluations, even if they must be conducted more often.
